# A salmonid EST genomic study: genes, duplications, phylogeny and microarrays

**DOI:** 10.1186/1471-2164-9-545

**Published:** 2008-11-17

**Authors:** Ben F Koop, Kristian R von Schalburg, Jong Leong, Neil Walker, Ryan Lieph, Glenn A Cooper, Adrienne Robb, Marianne Beetz-Sargent, Robert A Holt, Richard Moore, Sonal Brahmbhatt, Jamie Rosner, Caird E Rexroad, Colin R McGowan, William S Davidson

**Affiliations:** 1 Centre for Biomedical Research, University of Victoria, Victoria, British Columbia, V8W 3N5, Canada; 2 Genome Sciences Centre, BC Cancer Agency, Vancouver, British Columbia, V5Z 4S6, Canada; 3 Prostate Centre, Vancouver, British Columbia, V6H 3Z6, Canada; 4 ARS, USDA, Natl Ctr Cool & Cold Water Aquaculture, Kearneysville, WV 25430, USA; 5 Molecular Biology and Biochemistry, Simon Fraser University, Burnaby, British Columbia, V5A 1S6, Canada; 6 Department of Biology, University of Victoria, P.O. Box 3020, Victoria, British Columbia, V8W 3N5, Canada

## Abstract

**Background:**

Salmonids are of interest because of their relatively recent genome duplication, and their extensive use in wild fisheries and aquaculture. A comprehensive gene list and a comparison of genes in some of the different species provide valuable genomic information for one of the most widely studied groups of fish.

**Results:**

298,304 expressed sequence tags (ESTs) from Atlantic salmon (69% of the total), 11,664 chinook, 10,813 sockeye, 10,051 brook trout, 10,975 grayling, 8,630 lake whitefish, and 3,624 northern pike ESTs were obtained in this study and have been deposited into the public databases. Contigs were built and putative full-length Atlantic salmon clones have been identified. A database containing ESTs, assemblies, consensus sequences, open reading frames, gene predictions and putative annotation is available. The overall similarity between Atlantic salmon ESTs and those of rainbow trout, chinook, sockeye, brook trout, grayling, lake whitefish, northern pike and rainbow smelt is 93.4, 94.2, 94.6, 94.4, 92.5, 91.7, 89.6, and 86.2% respectively. An analysis of 78 transcript sets show *Salmo *as a sister group to *Oncorhynchus *and *Salvelinus *within Salmoninae, and Thymallinae as a sister group to Salmoninae and Coregoninae within Salmonidae. Extensive gene duplication is consistent with a genome duplication in the common ancestor of salmonids. Using all of the available EST data, a new expanded salmonid cDNA microarray of 32,000 features was created. Cross-species hybridizations to this cDNA microarray indicate that this resource will be useful for studies of all 68 salmonid species.

**Conclusion:**

An extensive collection and analysis of salmonid RNA putative transcripts indicate that Pacific salmon, Atlantic salmon and charr are 94–96% similar while the more distant whitefish, grayling, pike and smelt are 93, 92, 89 and 86% similar to salmon. The salmonid transcriptome reveals a complex history of gene duplication that is consistent with an ancestral salmonid genome duplication hypothesis. Genome resources, including a new 32 K microarray, provide valuable new tools to study salmonids.

## Background

Extensive knowledge of trout and salmon is a result of their widespread use in scientific research, as an environmental sentinel species and as a food and sport fish. Perhaps more is known about the physiology, ecology, genetics, behavior and biology of salmonids than any other fish group [1]. This background provides a wealth of data from an economically important and phylogenetically distinct group of fish that can help guide, and benefit from, new genomic studies.

The Salmonidae family includes: whitefish and ciscos (subfamily Coregoninae); graylings (Thymallinae); trout, salmon and charr (Salmoninae) [2]. Salmonids are classified into nine genera and sixty-eight species. They are native of the cooler climates of the Northern Hemisphere, but have been widely introduced around the world. Salmonids belong to a basal teleost Protacanthopterygii suborder (smelt, pike and salmon) group, which has been separated from other well studied euteleost lineages such as Ostariophysi (zebrafish, catfish, flathead minnow, etc.), and Acanthopterygii (cod, cichlids, fugu, sticklebacks, rockfish) for 217–290 MY [2-5].

The common ancestor of salmonids is purported to have experienced a whole genome duplication event between 25 and 100 MYA [6,7]. Extant salmonids are considered pseudo-tetraploid as they are in the later stages of reverting to a stable diploid state. Evidence for the ancestral salmonid autotetraploid genome duplication includes: multivalent chromosome formation during male meiosis and evidence for tetrasomic segregation at some loci [6]; one of the larger euteleost genome sizes (3–4.5 pg) with double that of sister groups Esociformes (0.8–1.8 pg, pike) and Osmeriformes (0.7 pg, smelt) [8]; homeologous chromosomal segments based on recent genetic maps and comparative studies using microsatellite markers, and duplicated gene family studies such as Hox, Major Histocompatibility complex (MH), growth hormone, and nineteen allozymes [6,9-12].

The genome duplication in salmonids is the most recent genome duplication in this lineage. There are now a number of studies and good evidence, primarily from sequenced zebrafish and pufferfish genome sequences, for tetraploidization/rediploidization early in the ray-finned fish lineage (350–400 MYA) [13-16]. Several of these studies have suggested that the ancestral fish duplication, in addition to the two ancestral vertebrate genome duplications, are part of the reason why ray-finned fishes make up nearly half of all extant vertebrates species and exhibit tremendous biodiversity affecting their morphology, ecology, behavior and evolution.

Vertebrate species diversity and body plan diversity have commonly been linked to genome duplications, although there is some debate on how well we can draw these conclusions based on the very old genome duplications commonly studied. Mechanistically, how a genome reorganizes itself to cope with duplicated chromosomes, gene dosage effects, and the role of gene duplications for evolution and adaptation are long-standing issues in biology that remain unresolved [6,13-17]. The number and diversity of salmonid species, and their relatively recent genome duplication, make salmonids ideal for examining recent events that could have played such a pivotal role in generating gene diversity and species diversity found in modern vertebrates.

The genomics resources of salmonids are being rapidly expanded through a few large-scale genomics programs [18-23]. Here we identify 354,061 new ESTs from Atlantic salmon and several other salmonid and related species in order to obtain a comprehensive view of the salmonid transcriptome, identify species relationships, identify gene duplications and introduce a new 32 K microarray tool for transcriptome analysis.

## Results and discussion

### cDNA libraries

New, directionally cloned, mixed tissue (brain, kidney and spleen), normalized cDNA libraries were constructed for Atlantic salmon (*Salmo salar*; European McConnell, and Canadian, Saint John River strains), chinook salmon (*Oncorhynchus tschawytscha*), sockeye salmon (*Oncorhynchus nerka*), brook trout (*Salvelinus fontinalis*), lake whitefish (*Coregonus clupeaformis*), grayling (*Thymallus thymallus*), and northern pike (*Esox lucius*). Separate normalized libraries were constructed from *Salmo salar *thymus, thyroid, and head kidney tissues. In addition, one full-length, mixed tissue, large insert (> 2 kb), non-normalized library was constructed to identify longer gene transcripts. cDNA clones were isolated, purified and sequenced from the 5' and 3' ends. Clone numbers and insert sizes for the different libraries and species that were done as part of this study are listed in Table 1.

**Table 1:** Salmonid cDNA libraries, sequencing and assembly summary statistics for data provided in this study.

**Species/Tissue/(library)**	**#** **clones**^a^	**Insert** **size**^b^	**#** **seq**^c^	**#** **contigs**^d^	**#. of** **singlets**^e^	**Max.** **contig**^f^	**Ave.** **contig**^g^	**% new** **(sp.)**^h^
***Salmo salar ***								
Thymus (evd)	31488	1.5	59264	23768	8685	66	2.3	16
Thyroid (eve)	30720	1.9	58700	28045	12378	37	2.1	15
Head kidney (evf)	31104	1.5	59541	28316	10832	30	2.1	16
Pyloric Caecum (pla, plb, plc, plna, plnb, pha, phc)	9584	0.9	13543	5691	2766	35	2.3	17
Brain, kidney, spleen (rgb2)	60288	1.6	97171	42562	26504	58	2.1	15
Brain, kidney, spleen (sjb)	5835	1.8	10085	6656	3541	8	1.5	18
								
***Oncorhynchus tschawytscha***								
Brain, kidney, spleen (rgd)	3840	2.1	5935	3941	2970	31	1.5	82
Brain, kidney, spleen (evc)	3744	1.8	5729	3841	2487	10	1.5	80
								
***Oncorhynchus nerka***								
Brain, kidney, spleen (rge)	7296	2.0	10813	6123	3924	173	1.8	98
								
***Salvelinus fontinalis***								
Brain, kidney, spleen (evi)	5376	1.4	10051	5424	1247	9	1.9	100
								
***Coregonus clupeaformis***								
Eye, kidney, spleen (evb)	4800	1.6	8630	5537	3359	12	1.6	93
								
***Thymallus thymallus***								
Brain, kidney, spleen (evl)	5760	1.5	10975	5926	1309	6	1.9	100
								
***Esox lucius***								
Brain, kidney, spleen (bkhp)	2304	0.9	3624	2420	1346	6	1.5	100

^a ^number of clones from which at least one sequence (5' or 3') was obtained

^b ^average EST fragment size cloned (kb), estimated from > 30 clone digests.

^c ^number of 5' and 3' EST sequences obtained

^d ^number of EST contigs (1^st ^stage assembly) that includes singlets

^e ^number of contigs containing a single sequence

^f ^the size of the contig containing the largest number of sequences

^g ^the average size of all contigs (includes singletons)

^h ^percent of the putative transcripts that are unique to the species.

### Transcript analysis: sequence and assembly

To obtain a comprehensive list of genes in salmonids, we used a strategy of deep 5' and 3' EST sequencing from a few high quality libraries. This approach complements previous studies, which examined more limited EST surveys of cDNA libraries from a large number of different tissues and developmental stages [18-22]. For Atlantic salmon, over 30,000 clones were sequenced from each of the thymus, thyroid, and head kidney tissue libraries. From previously described normalized libraries, [19] 9,584 additional clones were sequenced from the Atlantic salmon pyloric caecum tissue library and 60,288 additional clones were sequenced from a mixed tissue library (rgb2; Table 1). The total number of clones examined from the rgb2 library was 84,176 which yielded 127,660 sequence reads or 30% of the total Atlantic salmon EST database. Even with this deep sequencing, nearly 13% of the last 637 reads were novel (< 99% over 100 bp) and the maximum redundancy for a single transcript from the rgb2 library was 58 (Table 1).

The results of the assembly of 298,304 Atlantic salmon ESTs obtained in this study along with 138,325 ESTs from previous studies [[19,22], GenBank] are shown in Table 2. Due to the complexities of the salmonid genome duplication and because it provides a stable, conservative starting point for all subsequent analyses, our analysis began with a first stage assembly using stringent parameters (PHRAP: 0.99 repeat stringency and 100 minscore). A second stage assembly (96% repeat stringency and 300 minscore) was implemented to combine some of contigs which may be alleles, or possibly very recent gene duplications (distinguishing among alleles, minor assembly errors, miss-calls and very recent gene duplications, particularly in lower quality sequence regions is very difficult in the absence of genomic sequence data). In Atlantic salmon, 81,398 potential transcripts (2 stage assembly) were identified, of which 29,844 (37%) were similar (BLASTX, 1e-10) to annotated sequences in CDD or SwissProt protein databases. For comparison, an assembly of 246,704 ESTs from rainbow trout [[18,21], GenBank] resulted in 51,199 transcripts, of which 19,266 (38%) had BLASTX hits. Assembled contigs are available [24].

**Table 2:** Summary of salmonid ESTs and contig assemblies.

	**Atlantic** **salmon**	**Rainbow** **trout**	**Chinook** **salmon**	**Sockeye** **salmon**	**Brook** **trout**	**Lake** **whitefish**	**Grayling**	**Northern** **pike**	**Rainbow** **Smelt**
# EST sequences^a^	436629	246704	14535	12056	10051	10842	10975	3624	36785
									
Assembly Stage1^b^									
# contigs (2+)^c^	70,845	42423	2890	2480	4178	4464	4616	1074	9044
# singletons^d^	47,139	26935	6295	4118	1247	2510	1314	1346	7019
# transcripts^e^	117,984	69358	9185	6598	5425	6974	5930	2420	16063
									
Assembly Stage2^f^									
# transcripts^g^	81398	51199	8517	6200	4946	6446	5408	2380	12159
# hits^h^	29844	19266	3684	3561	1838	2314	1780	198	6139
% with hits^i^	37	38	43	57	37	36	33	8	50

^a ^number of EST sequences for all of the species including those in GenBank

^b ^Assembly stage 1 refers to PHRAP assembly using parameters 0.99 repeat_frequency and 100 minscore

^c ^number of contigs with 2 or more sequences

^d ^number of contigs with 1 sequence

^e ^total number of transcripts including singletons

^f ^Assembly stage 2 refers to PHRAP assembly using parameters 0.96 repeat_frequency and 300 minscore

^g ^the number of transcripts that result from a re-assembly of all stage 1 transcripts using PHRAP parameters 96 repeat_frequency and 300 minscore

^h ^number of transcripts that have a BLASTX hit of < 1e-10 to SwissProt/CDD databases.

^i ^percent of stage 2 assembled transcripts that have a BLASTX hit.

Transcript surveys of additional salmonid species included 4,800–7,500 clones sequenced from each of chinook salmon, sockeye salmon, brook trout, grayling and lake whitefish. 11,664 sequences were obtained from chinook salmon, 10,813 sequences from sockeye salmon, 10,051 sequences from brook trout, 10, 975 sequences from grayling and 8,630 sequences from lake whitefish. Sequence, assembly and summary statistics are shown for those data obtained in this study (Table 1) and when combined with data from public databases (Table 2). In addition, to provide non-genome-duplicated sister group comparisons, 2,304 clones were sequenced from northern pike (3,624 sequences) (Table 1 and 2). For many of these species, the ESTs provided in this study represent nearly all or most of the known transcripts. Recently published data from rainbow smelt (*Osmerus mordax*) [23] was also included in Table 2.

**Figure 1:**
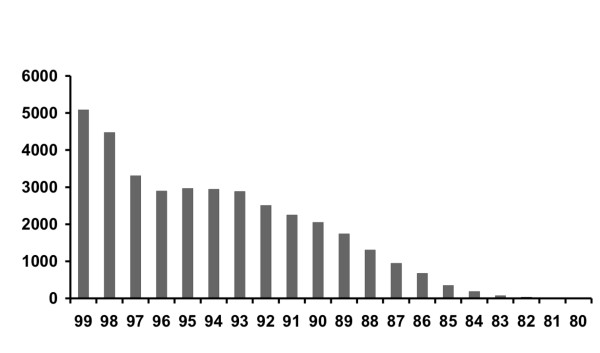
Number of aligned contigs (y-axis) out of 81,398 total contigs is plotted against percent similarity of alignments (x- axis).

To examine the relationships among the contig consensus sequences of Atlantic salmon we compared all contigs (including singletons) against each other by BLAST and plotted the number of top pair-wise alignments (E-value < 1e-50; length > 200 bp) with the identity score (Figure 1). 36,775 contigs showed greater than 80% identity over 200 bp to at least one other contig. Of these, 12,883 were 97–99.9% similar to at least one other contig. These contigs may represent alleles, recent duplicates or errors in sequence data. 23,892 contigs show between 80 and 96.9% identity with at least one other contig. The large number of duplicated transcripts observed in the Atlantic salmon genome is consistent with the hypothesis of an ancestral salmonid genome duplication, though it is surprising that so many of the duplicated contigs are so similar. This observation is being pursued further in a separate study. The analysis of contig similarity shows that the majority of the 81,398 contigs represent distinct transcripts. Note that since the assembly process itself combines sequences with high levels of similarity (> 96% repeat stringency with minscore > 300; see Methods), very recent duplications may not all be identified in this process. Furthermore, since the species used in this study differ by greater than 5% (Table 3), this process would be expected to identify ancestral salmonid duplications occurring at or prior to the rainbow trout and Atlantic salmon speciation.

**Table 3:** Cross-species comparisons of contig transcripts.

	# contigs	# missing in AS^a^	# missing in RT^b^	# missing in both^c^	% sim to AS^d^	Avelen^e^	% sim to RT^f^	Avelen
Atlantic salmon (SJ)^g^	5781	479	1210	354	98.4	705	93.4	493
Atlantic salmon (all)^h^	81398	*na*	36351	*na*	*na*	*na*	93.3	504
Rainbow trout	50256	13626	*na*	*na*	93.8	495	*na*	*na*
Chinook salmon	8517	797	1224	426	94.2	510	95.5	510
Sockeye salmon	6200	577	770	298	94.6	571	95.7	569
Brook trout	5424	285	627	174	94.4	580	93.9	522
Lake whitefish	6446	804	1420	608	92.5	425	92.2	399
Grayling	5408	657	1136	506	91.7	435	91.3	400
Northern pike	2380	1894	2001	1846	89.6	241	89.4	251
Rainbow smelt	12159	7462	7812	6920	86.2	431	86.1	419

^a ^number of contigs that are not found in the Atlantic salmon database

^b ^number of contigs that are not found in the rainbow trout database

^c ^number of contigs that are not found in either the Atlantic salmon or rainbow trout database

^d ^percent identity compared to the top BLASTN hit to the Atlantic salmon database over 200 bp and e-value < 1e-25. In the case of Atlantic salmon (SJ) the comparison is to the McConnell strain.

^e ^average length of the BLASTN hit

^f ^percent identity compared to the top BLASTN hit to the rainbow trout database over 200 bp and e-value < 1e-25

^g ^only Atlantic salmon ESTs from the Saint John River strain

^h ^all Atlantic salmon ESTs other than those in note "g" above

Determining the number of genes in Atlantic salmon from the number of EST contigs is difficult for several reasons; 1) the partial representation of genes by EST sequences may result in several contigs associated with a single gene transcript, 2) allelic or recently duplicated genes may be represented by similar but unique transcripts (this latter case is particularly problematic in pseudotetraploid salmonids), 3) alternative splicing, alternative poly adenylation and termination sites from the same gene can result in different transcripts, and 4) transcription products can occur from intergenic regions. An estimation of the number of genes in salmonids will require additional information such as full-length cDNA sequences and gene mapping information.

### Salmonid comparisons

Similarity among the different salmonid species was assessed using the top BLASTN hit against Atlantic salmon and rainbow trout EST contig databases. The similarity values from chinook salmon, sockeye salmon, rainbow trout, Atlantic salmon (McConnell and Saint John River strains), brook trout, grayling, lake whitefish, northern pike and rainbow smelt are shown in Table 3. Assembled contigs (2-stage), rather than individual reads were used for all comparisons to reduce the impact of redundant transcripts. Chinook, sockeye, brook trout, grayling and lake whitefish average 95.5, 95.7, 93.9, 91.3 and 92.2% identity to rainbow trout, and 94.2, 94.6, 94.4, 91.7 and 92.5% identity to Atlantic salmon with over 87% of the contigs matching (E-value < 1e-25) at least one contig in the rainbow trout or Atlantic salmon databases. These comparisons provide only a very general indication of the similarity between transcriptomes of various salmonids, as assemblies contain both 5' (generally genic regions) and 3' (generally 3'-UTR regions) transcript reads. However, these DNA sequence similarity values correspond well to the limited number of values in the literature. Non-coding sequence similarity between rainbow trout and Atlantic salmon are 95% over 120 kb in MH class IA and B loci [12], and 93–97% over 4 kb in growth hormone (GH) genes [11]. Similarity between salmon and whitefish is 90–93% in GH genes [11].

Northern pike and rainbow smelt average 89.4 and 86.1% identity to rainbow trout and 89.6 and 86.2% identity to Atlantic salmon, but only 25–39% of these contigs matched anything in the rainbow trout or Atlantic salmon database. These latter comparisons have many fewer significant similarities identified partly because of the much older divergence times [3]. However, the reason for the lower than expected number of matches between northern pike and rainbow trout or Atlantic salmon is not clear. While the more distantly related rainbow smelt contigs show similar numbers of BLASTX hits to protein databases as salmonids, the northern pike contigs showed very few similarities to Atlantic salmon and rainbow trout contigs (25% compared to 39% for rainbow smelt and 87% for lake whitefish) and very few BLASTX hits to protein databases (8% compared to 50% for rainbow smelt and 36% for lake whitefish). One possible explanation may be due to longer 3'-UTRs in northern pike, but this remains to be confirmed.

### Transcriptome representation

It is difficult to assess how comprehensive the extensive Atlantic salmon and rainbow trout EST databases are. However, 73% (37,573 of the 51,199) of all rainbow trout contigs are also found in Atlantic salmon. Moreover, only 28% of those transcripts unique to rainbow trout (13,626) have protein hits (E < 1e-25) that support their legitimacy as genic regions, while other single ESTs may be from spurious transcription. 91% of lake whitefish transcripts have a significant similarity (BLASTN comparisons with e-values less than 1e-25) to the Atlantic salmon or rainbow trout databases. Comparative data from chinook salmon, sockeye salmon, brook trout, grayling, lake whitefish and rainbow smelt are provided in Table 3. Overall, these data provide support for extensive gene coverage in salmonid EST databases.

### Full-length analysis

The rapid progress of EST sequencing has enabled an estimation of the number of full-length cDNA clones. Full-length cDNAs (fl-cDNAs) are defined as having a "Start – Open Reading Frame (ORF) – Stop – 3' UTR – polyA signal" with the ORF corresponding to a full-length protein. Given multiple start and stop sites, alternative splicing and partial homologies to known proteins, it is difficult to give precise numbers of completed fl-cDNAs. However, TargetIdentifier (using BLAST comparisons to full-length genes in databases and Start signals; [25]) identifies 17,399 possible fl-cDNAs (averaging 1,361 bp in length) from the 81,398 possible transcripts in Atlantic salmon and 10,453 fl-cDNAs from the 51,199 rainbow trout transcripts. Thus far, about half of the predicted fl-cDNA meet all of the criteria above, and many of the fl-cDNAs are already fully characterized on a single clone. These tend to be the shorter (< 1.5 kb) genes. The list of over 10,000 putative fl-cDNA transcripts assembled from ESTs is available at the GRASP website [24] and further identification of clones for complete sequence analysis is underway.

### Salmonid EST, assembly, ORF and annotation database

**Figure 2:**
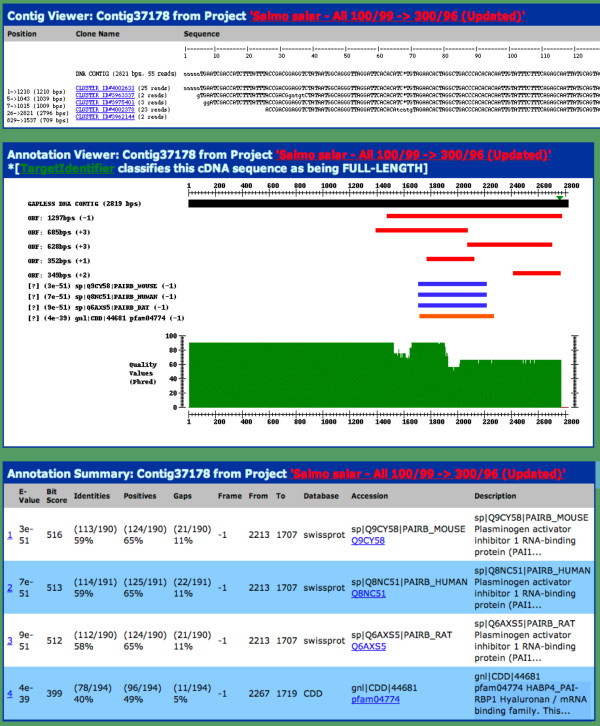
**Screen shot of Atlantic salmon contig viewer. **The top panel shows the alignment of 100/99 (first stage) clusters along with the number of individual EST reads in each. The second panel shows the 5 largest ORFs and reading frame, the BLASTX hits and reading frame, the Phred quality scores for each aligned position, and indicates whether TargetIdentifier has indicated that this clone is full-length and the predicted position of the START codon (green triangle). Selectable colored bars provide alignment links. The third panel gives specifics of the database hits and links to alignments and database entries.

All ESTs have been deposited in GenBank, however the EST assemblies themselves and the resulting consensus sequences are also very useful in identifying genes. These assemblies, together with the raw data are available [24]. The assembly consensus sequences are available for download and for searching using BLAST tools. A contig visualization tool was developed to allow users to search for similar consensus sequences using BLAST searches, identifying consensus names and then visualizing the sequences, alignment, open-reading frames (ORFs), TargetIdentifier predictions, and BLASTX hits in a single view (Figure 2: Cluster tools). Until such time as the genomes are completed, this database provides the salmonid community with access to several levels of EST and gene analyses.

### Salmonid phylogeny and gene duplication

**Figure 3:**
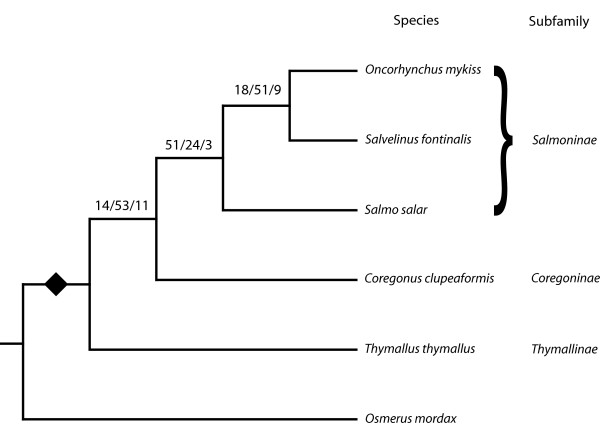
**Summary of 78 gene set consensus (70%) trees depicting the relationships among the major groups of Salmonidae.** Each branch shows the number of consensus trees supporting the branch, the number of trees providing no information and the number of trees contradicting the branch. The diamond at the base of the Salmonidae cladogram indicates the position where the majority of gene duplications were identified. The individual gene trees that pertain to each branch position are indicated in Table 4.

The relationships among major groups of salmonids have been largely unresolved, particularly with respect to the placement of *Salvelinus *(represented in this study by brook trout), *Oncorhynchus *(represented here by rainbow trout, chinook and coho salmon) and *Salmo *(Atlantic salmon) within Salmoninae, and the placement of Thymallinae (grayling), Coregoninae (whitefish) and Salmoninae (salmon) within Salmonidae [2,11,26-30]. From the EST contigs (Table 2), 78 separate gene sets have been identified, each of which contained at least one EST contig sequence from each of five major salmonid genera (*Oncorhynchus, Salmo, Salvelinus, Coregonus, and Thymallus*), in addition to representation by a non-salmonid (*Osmerus*). Contig sequences within each gene set were aligned, trimmed to a common length (minimum of 300 bp) and analyzed using phylogenetic methods. 73 of the 78 gene sets could be identified by BLASTX searches to SwissProt databases (Table 4). For each gene set, a 70% neighbour-joining (NJ) consensus tree based on 500 bootstrap replicates was generated and the consensus tree rooted with *Osmerus mordax *sequences (rainbow smelt). The single species tree shown in Figure 3 represents a compilation of the phylogenetic results from 78 gene sets. In the summary tree, each branch is noted by; i) the number of 70% consensus NJ trees supporting the branch ii) the number of 70% consensus trees providing no resolution to the branch point, and iii) the number of consensus trees that conflict with the shown result. In this summary, the placement of *Salmo *as a sister group to *Oncorhynchus *and *Salvelinus *is supported in 18 of the 27 gene consensus trees for which resolution was found. Eight alternative consensus trees support grouping *Salmo *and *Salvelinus*, one consensus tree supports grouping *Salmo *and *Oncorhychus*, and the remaining 51 trees provide no resolution. Thus the overall result is in agreement with some of the more recent studies examining mitochondrial and nine nuclear genes [26], and suggests good support for grouping *Oncorhynchus *and *Salvelinus *apart from *Salmo *within the Salmoninae subfamily.

**Table 4:** Gene sets used in phylogenetic analysis.*

**Tree**	**# of Contigs**	**Align Length**	**Salmon Group**	**SubfamilyGroup**	**Duplication Salmonidae**	**Gene Description**		
						
						**Accession**	**e-value**	**SwissProt Description**
1	25	302	Om/Sf	-	no	Q9EPH8	0.0E+00	Polyadenylate-binding protein 1
2	11	287	-	-	yes	Q92572	1.0E-103	AP-3 complex subunit sigma-1
3	18	455	Om/Sf	-	yes	P41134	4.0E-31	DNA-binding protein inhibitor ID-1
4	16	283	-	-	yes	Q24117	2.0E-46	Dynein light chain 1, cytoplasmic
5	11	438	Om/Sf	C/T	yes	P38400	0.0E+00	Guanine nucleotide-binding protein G(i)
6	26	271	-	-	-	P09486	1.0E-138	SPARC precursor
7	12	301	-	-	no			Unknown
8	17	307	Om/Sf	-	yes	P62161	4.0E-80	Calmodulin
9	12	341	Ss/Sf	-	no	Q9Y5S9	4.0E-80	RNA-binding protein 8A
10	16	370	Om/Sf	-	no	P51410	4.0E-95	60S ribosomal protein L9
11	11	305	Om/Sf	-	yes	Q3MHN0	3.0E-95	Proteasome subunit beta type-6 precursor
12	8	411	-	-	-	O60493	1.0E-82	Sorting nexin-3
13	7	448	Ss/Sf	-	yes	O15247	8.0E-97	Chloride intracellular channel protein 2
14	13	500	Om/Sf	-	no	Q9NPI5	6.0E-73	Nicotinamide riboside kinase 2
15	14	379	Om/Sf	S/C	no	P13668	2.0E-47	Stathmin
16	7	638	Ss/Sf	-	no	Q6IQU6	1.0E-154	Ribosome production factor 1
17	11	713	-	-	yes	Q9D915	6.0E-23	Uncharacterized protein C8orf4 homolog
18	9	438	-	-	-	Q8VHZ7	1.0E-125	U3 small nucleolar ribonucleoprotein
19	10	314	-	-	yes	Q05826	1.0E-35	CCAAT/enhancer-binding protein beta
20	18	313	-	-	yes	P97371	1.0E-68	Proteasome activator complex subunit 1
21	10	505	-	C/T	yes	Q96GG9	1.0E-135	DCN1-like protein 1
22	10	620	-	-	yes	Q3T0B6	1.0E-91	Complement 1 Q subcomponent-binding
23	12	442	-	S/C	-	P05141	1.0E-149	ADP/ATP translocase 2
24	8	517	-	-	no	Q9UM00	2.0E-78	Transmembrane and coiled-coil domain
25	14	471	Om/Sf	-	yes	Q6PC69	1.0E-101	60S ribosomal protein L10a
26	13	409	-	-	no	Q5RE33	2.0E-67	Receptor expression-enhancing protein 5
27	14	308	Om/Sf	-	yes	P50397	0.0E+00	Rab GDP dissociation inhibitor beta
28	19	311	Ss/Sf	S/C	yes	P30044	8.0E-64	Peroxiredoxin-5, mitochondrial prec.
29	6	428	-	S/C	no	P15156	1.0E-115	Calcium-dependent serine proteinase
30	10	355	-	S/C	yes			Unknown
31	15	486	-	-	yes	Q9CXL1	8.0E-78	Transmembrane protein 50A
32	11	332	-	-	yes	Q62636	2.0E-90	Ras-related protein Rap-1b precursor
33	22	291	Om/Sf	-	yes	Q3T0Q6	3.0E-71	Cellular nucleic acid-binding protein
34	10	268	-	-	yes	O54734	0.0E+00	Dolichyl-diphosphooligosaccharide
35	8	367	Om/Sf	S/C	no	Q9W719	1.0E-117	Hypoxanthine-guanine phosphoribosyltran.
36	9	609	Om/Sf	-	no	O42123	2.0E-48	FK506-binding protein 1A
37	7	379	-	-	yes	Q9Y5K5	1.0E-161	Ubiquitin carboxyl-terminal hydrolase
38	17	599	Ss/Sf	C/T	yes	P50897	1.0E-120	Palmitoyl-protein thioesterase 1 prec.
39	12	408	-	S/C	no	Q75AA8	7.0E-44	Translation machinery-associated protein
40	15	389	Ss/Sf	-	yes	Q9UL46	4.0E-76	Proteasome activator complex subunit 2
41	8	413	-	-	no	Q96A49	1.0E-100	Synapse-associated protein 1
42	20	333	-	S/C	yes	Q9JK11	8.0E-66	Reticulon-4
43	17	336	-	S/T	no	P67810	5.0E-97	Signal peptidase complex catalytic sub.
44	11	483	-	S/C	yes	Q5XIH7	1.0E-112	Prohibitin-2
45	7	419	Om/Sf	-	yes	P28497	1.0E-136	F-actin-capping protein subunit alpha-2
46	14	465	-	-	no	Q8BLR9	7.0E-16	Hypoxia-inducible factor 1 alpha inhibitor
47	9	610	Om/Sf	-	yes	O75940	9.0E-96	Survival of motor neuron-related-splicing
48	19	604	-	C/T	yes	P60517	4.0E-61	Gamma-aminobutyric acid receptor-
49	17	413	-	C/T	yes	Q6NUC2	1.0E-161	COP9 signalosome complex sub. 6
50	13	350	-	S/C	yes	Q28104	1.0E-141	Coatomer subunit epsilon
51	16	289	-	-	yes	Q2VIU1	7.0E-55	DNA-binding protein inhibitor ID-2
52	12	484	Om/Sf	S/C	yes	P30101	0.0E+00	Protein disulfide-isomerase A3 prec.
53	19	356	-	-	yes	Q6DH65	4.0E-81	Density-regulated protein
54	9	439	Om/Sf	S/C	yes	P26990	4.0E-99	ADP-ribosylation factor 6
55	8	300		-	yes	Q13491	1.0E-124	Neuronal membrane glycoprotein M6-b
56	10	273	-	-	yes	O93277	0.0E+00	WD repeat-containing protein 1
57	11	304	-	S/T	yes	P08132	1.0E-115	Annexin A4
58	12	562	-	-	no	Q12962	3.0E-63	Transcription initiation factor TFIID sub.
59	7	553	-	-	yes	Q9NS69	2.0E-18	Mitochondrial import receptor subunit
60	8	561	Ss/Sf	S/C	no	Q6AYU1	1.0E-158	Mortality factor 4-like protein 1
61	11	378	-	-	no	P50169	1.0E-95	Retinol dehydrogenase 3
62	16	509	-	-	yes	P59998	2.0E-87	Actin-related protein 2/3 complex sub. 4
63	7	619	-	-	no			Unknown
64	12	310	-	-	no	P62316	4.0E-52	Small nuclear ribonucleoprotein Sm D2
65	10	398	-	-	yes	P16527	8.0E-23	Myristoylated alanine-rich C-kinase sub.
66	10	277	-	S/T	yes			Unknown
67	7	388	-	-	yes	O54968	3.0E-80	Nuclear factor erythroid 2-related factor 2
68	8	311	-	-	no	P22232	1.0E-124	rRNA 2'-O-methyltransferase fibrillarin
69	14	300	Om/Sf	-	yes	P40926	1.0E-157	Malate dehydrogenase, mito. prec.
70	9	589	-	-	yes	Q64422	1.0E-138	Glucosamine-6-phosphate isomerase
71	13	577	Ss/Sf	S/C	yes	Q58DU5	1.0E-127	Proteasome subunit alpha type-3
72	14	210	-	C/T	yes	Q15008	0.0E+00	26S proteasome non-ATPase reg. sub.
73	12	348		C/T	no			Unknown
74	18	409		-	yes	Q16799	7.0E-77	Reticulon-1
75	19	462		-	yes	Q9D1J3	5.0E-39	Nuclear protein Hcc-1
76	8	621		-	yes	P19387	1.0E-145	DNA-directed RNA polymerase II subunit
77	16	482		-	yes	Q02878	1.0E-106	60S ribosomal protein L6
78	11	552		C/T	yes	Q802F2	3.0E-95	Selenoprotein T1a precursor

* Listed is the gene set identifier (tree number) along with the number of contigs used in each data set, the length of the respective nucleotide alignment (no gaps), and tentative identification based on BLASTX hits to the SwissProt database (accession number, E-value and description). For each gene set, the tree support for the various arrangements is listed; for example, Om/Sf supports an *Oncorhynchus mykiss/Salvelinus fontinalis *grouping; or S/C supports a Salmoninae/Coregoninae grouping. In addition there is an indication whether a tree is consistent (yes/no) with an ancestral Salmonidae gene duplication. "-" indicates that the data provides no clear evidence for any particular tree. All EST accession numbers used to make contig consensus sequences, all alignments and the 70% consensus trees are available [see Additional file 1] or online at the GRASP website [24].

Consistent with traditional nomenclature, the Salmoninae group, which includes *Salvelinus*, *Oncorhynchus *and *Salmo *is also very well supported with 51 of the 54 resolved trees consistent with this grouping. The three discrepant trees supported a *Salmo/Coregonus *grouping.

The relationships among the three subfamilies within Salmonidae have not been extensively addressed at the molecular level. However, on the basis of a morphological analysis, Coregoninae (whitefish and ciscos) has been hypothesized as the earliest branch within the salmonids [[29], also see [30]]. In the present analysis, 14 of the 25 informative gene sets are more consistent with the basal position of Thymallinae (Figure 3). Of the discrepant trees, 8 sets support a Thymallinae/Coregoninae grouping and 3 support an ancestral position of Coregoninae. While these data are not definitive, there appears to be some support for an ancestral Thymallinae branching within the Salmonidae with Coregoninae as the sister group to Salmoninae. These data provide the first large-scale molecular view of salmonid subfamily relationships and provide an important perspective on future analyses of duplicated genes, as well as physiological and ecological traits [27] that have evolved subsequent to the ancestral salmonid genome duplication.

The salmonid whole genome duplication hypothesis makes it is difficult to separate an analysis of species relationships from gene phylogeny. One expectation arising from a relatively recent genome duplication is evidence for extensive nuclear gene duplicates. Subsequent to the genome duplication, the number of observed duplicated transcribed genes is expected to decrease as, over time, one of the duplicates becomes transcriptionally inactive. When multiple species are examined, some species may have both duplication products while other species may have only one representative. Evidence of an ancestral duplication is identified in gene trees that contain multiple species trees that may have missing representatives. Of the 78 gene sets examined in this study, 51 show clear evidence of multiple species trees within gene trees that are consistent with a gene duplication in the ancestor of Salmonidae, sometime after the separation of Osmeriformes and Salmoniformes fish. 23 gene sets (Table 4) provided no evidence for any ancestral gene duplication, and 4 sets could not be interpreted. The data from 78 gene sets representing 372 consensus sequences and 11,397 bp of aligned DNA from five salmonid genera, indicate that a large number of salmonid genes show evidence of extensive gene duplication at a phylogenetic position that is consistent with the whole genome duplication in the ancestral Salmonidae hypothesis. Further studies of Esociformes fish will more precisely establish the timing of some of these gene duplications.

### Salmonid 32 K microarray

To use the data generated by ESTs and assemblies for examining gene expression, a new 32,000 feature cDNA microarray was developed. This new array is based on the existing 16 K GRASP array [31] plus 14,496 additional Atlantic salmon and 1,491 additional rainbow trout contigs that were identified as unique and were successfully amplified in this study. The 32 K cDNA microarray is composed mainly of 27,917 Atlantic salmon (AS) and 4,065 rainbow trout (RT) cDNA elements or features. 54% of the elements have fairly stringent (1-e10) hits to annotated members in public protein databases. Hybridization performance of this array was evaluated using Atlantic salmon, rainbow trout, coho salmon, brook trout and lake whitefish RNA obtained from liver organs. The success of hybridization of labeled target to the salmonid elements was judged by the numbers of Atlantic salmon and rainbow trout elements passing background plus 2 SD threshold values (see Methods). No transformations or normalizations were performed on the data. Overall statistics are presented in Table 5. In summary, for RNA isolated from the liver of Atlantic salmon, rainbow trout, coho salmon, brook trout and lake whitefish, an average of 48% of the 32,018 elements showed significant detection levels of expression. Comparing these results to that from the previous 16 K GRASP arrays indicates that doubling the number of elements from 16 K to 32 K resulted in the ability to assess expression patterns of approximately 61% additional transcripts. This represents a substantial increase in our ability to assess gene transcription patterns in salmonids. The hybridization performances of the different salmonid species (assessed from numbers of Atlantic salmon and rainbow trout elements passing threshold) conformed to expectations, given the close evolutionary relationships of the species tested (92–94% identity, Fig. 3) and, with the possible exception of brook trout, all members of the family Salmonidae tested showed similar levels of hybridization to the Atlantic salmon and rainbow trout elements on the 32 K microarray. As the Salmonidae family represents 68 closely related species, the 32 K cDNA array provides an excellent opportunity to evaluate gene expression patterns of a large group of culturally and economically important species.

**Table 5:** Cross species hybridization results for the salmonid 32 K cDNA microarray. *

Salmonid Species	% +'ve	%CV
Atlantic salmon (n = 4)	48.6%	12.0%
Rainbow trout (n = 4)	58.1%	9.8%
Coho (n = 4)	52.3%	23.2%
Brook Trout (n = 4)	35.0%	2.4%
Whitefish (n = 4)	47.7%	7.8%

* Percent elements on cDNA array with median signal intensity greater than threshold (background signal+ 2SD). %CV is percent coefficient of variation and "n" is the number of biological replicates.

## Conclusion

Atlantic salmon and rainbow trout now rank 19^th ^and 29^th ^in terms of species representation in EST databases with over 730,000 salmonid ESTs in total. Almost half of these data are presented in this study. These data provide an excellent genetic resource for physiological, ecological, biochemical, behavioral, disease and biological studies of salmonids. They also provide key materials for the development of polymorphic markers for genetic and physical genomics maps, for the identification and analysis of proteins and for the development of microarrays and primers for transcriptional analyses.

Transcript assemblies and analyses have identified over 81,000 possible transcripts from Atlantic salmon and 51,000 transcripts from rainbow trout. These assemblies and consensus sequences are available from the author or through a database housed on the GRASP website [24]. As many as 17,399 full-length *Salmo salar *gene assemblies are present in this database.

Comparison of orthologous ESTs from Atlantic salmon, rainbow trout, chinook salmon, sockeye salmon, brook trout, lake whitefish, grayling, northern pike and rainbow smelt show that Pacific salmon (*Oncorhynchus*), Atlantic salmon (*Salmo salar*) and brook trout (*Salvelinus fontinalus*) average 94–96% similarity. Lake whitefish (*Coregonus clupeaformis*) and grayling (*Thymallus thymallus*) are more distant from Pacific and Atlantic salmon (92%), followed by northern pike (*Esox*; 89%) and rainbow smelt (*Osmerus*; 86%). A view of salmonid relationships and support for the salmonid genome duplication has been found. With the new EST database, a new, more extensive 32 K cDNA microarray has been developed to help assess gene expression patterns in salmonids.

## Methods

### Tissues, RNA, Aquaculture and Sampling

*Salmo salar *(McConnell strain), *Oncorhynchus tshawytscha *and *Oncorhynchus nerka *tissues were obtained from the Department of Fisheries and Oceans (Robert Devlin, WestVan Lab., West Vancouver, British Columbia). *Salvelinus fontinalis *and *Coregonus clupeaformis *tissues were obtained from Louis Bernatchez (Laval University, Quebec). *S. salar *(Saint John River strain; brain, kidney and spleen) were obtained from Vanya Ewart (NRC Institute for Marine Biosciences, Nova Scotia). *Thymallus thymallus *brain, kidney and spleen tissues were obtained from Craig Primmer (University of Turku, Finland). *Esox lucius *were captured by gill net from Charlie Lake British Columbia. All fish were euthanized, followed by rapid dissection of tissues. Tissues were flash frozen in liquid nitrogen or dry ice and stored at -80°C until RNA extraction.

### cDNA libraries

Total RNA or poly(A)+ RNA (FastTrack MAG kit; Invitrogen) was extracted from flash frozen tissues. *Salmo salar *and *Oncorhynchus tshawytshca *mixed tissue (spleen, head kidney, brain) libraries were directionally constructed in both pCMV Sport-6.1 (Research Genetics Inc.) and pAL-17.3 (Evrogen Co.). *S. salar *(normalized head kidney, thymus and thyroid), *Coregonus clupeaformis. Thymallus thymallus *and *Salvelinus fontinalis *libraries were constructed in pAL-17.3 (Evrogen). The *Oncorhynchus nerka *mixed tissue normalized library was also constructed in pCMV Sport-6.1 (ResGen). *S. salar *(mixed tissue St. John strain) and *Esox lucius *libraries were constructed in pDNR-Lib using Creator SMART cDNA library construction kits (Clontech). Insert sizes of cDNA libraries were determined by visual comparison of clone restriction fragments with the DNA size markers *Hind*III (GibcoBRL) and 1 kb ladder (GibcoBRL).

### Sequencing, Sequence Analysis, and Contig Assembly

Clone libraries were plated and robotically arrayed in 384-well plates. Glycerol stocks of overnight cultures were prepared in 384-well format [19]. Plasmid DNAs were extracted and BigDye™ Terminator (ABI) cycle sequenced on ABI 3730 sequencers using conventional procedures and the following primers: 5'-T_18_-3', M13 forward (5'-GTAAAACGACGGCCAGT-3'), M13 reverse (5'-AACAGCTATGACCAT-3' or 5'-CAGGAAACAGCTATGAC-3') and for the Evrogen libraries SP6WAN primer was used for the 3' end sequencing. Base-calling from chromatogram traces was performed using Phred [32,33]. Vector, poly-A tails, and low quality regions were trimmed from EST sequences; sequences that had less than 100 good quality bases after trimming were discarded [19]. Initial assembly of ESTs into contigs used PHRAP [34], under stringent clustering parameters (minimum score: 100; repeat stringency: 0.99). Contig consensus sequences and singleton sequences were aligned with non-redundant GenBank nucleotide and amino acid sequence databases (SwissProt, PBL, CDD, and UniRef90) using BLASTN or BLASTX [35-37]. Sequence databases, assemblies, consensus sequences, tools such as BLAST and RepeatMasker [38], and sequence and consensus annotations are freely available from the author and from the GRASP website [24].

The number of *Salmo salar *contigs was assessed using the PHRAP assembly program because of its ability to assemble very large numbers of ESTs in a single run, and its integration with PHRED base quality scores on primary reads and subsequent consensus sequences. The CAP3 assembler [39] was also used and similar results were obtained for smaller datasets. For this study, contig assembly employed a two-stage process. The first stage assembly used parameters 100 minscore and 0.99 repeat stringency to build contigs and consensus sequences that appeared to separate alleles of many transcripts. The second stage used the consensus sequences (with quality scores) from the first stage and parameters 96% repeat frequency and 300 minscore to build contigs and consensus sequences that appeared to combine some of the contigs that contained some base calling discrepancies, as well as what appeared to be alleles or very recently duplicated genes. Various parameters were tested and final parameters were chosen to minimize the number of contigs, where the number of contigs changed the least with respect to small changes in parameter values, and where distinct contigs appeared to have some biological significance (i.e., 99/100 appeared to separate many alleles and 96/300 as a second stage appeared to join some alleles and provided values that separated a clear majority of orthologous salmonid gene comparisons). With both sets of parameters, we were able to discriminate between similar sequences from different salmonid species. Sequences in contigs containing more than one polyA site were removed from the assemblies as they may represent chimeric clones.

Assemblies provide rough estimates of transcripts. Several algorithms have been examined and all have strengths and weaknesses. Examples of other assemblies include DFCI gene indexes [40,41] that estimate 83,554 TCs+singletons from 244,984 rainbow trout ESTs and 63,138 contigs from a partial 236,009 EST dataset from Atlantic salmon (these assemblies are periodically updated). INRA [21] using CAP3 estimates 56,392 transcripts (contigs + singlets) from 326,719 rainbow trout ESTs and 45,349 contigs from a partial Atlantic salmon EST database. UniGene [42], from NCBI does not provide true assemblies and may cluster duplicated genes into single bins, which is problematic in salmonids. UniGene estimates approximately 30,000 and 25,000 UniGene sets in Atlantic salmon and rainbow trout respectively. While differences exist, the general number of estimated transcripts is similar. Problem areas that have been identified in assemblies tend to be associated with long transcripts, so these contigs will have to be treated carefully and perhaps manually edited. Assemblies are freely available from the author and the GRASP website [24]. As a caveat, because of the purported recent duplication of the salmonid genome and potential for miss-assembly of duplicated transcripts, these contigs have to be treated with caution.

Percent identity measures between contig consensus sequences from the various species were obtained from BLASTN alignments where a minimum length of 200 bp was observed. As in other distance measures, this finds the most similar sequence fragments and is biased high, particularly for more distant comparisons. A partial estimate of the impact on more distantly related sequence comparisons is the increased number of contigs for which no cross-species alignments were found and the reduction in average length of alignments. These values are provided in Table 3. However, the percent identity measure provides an estimate of observed similarity that is useful for evaluating potential cross-species DNA hybridizations in microarray experiments (see below).

### Gene phylogenetic analysis

Contig sequences from *Salmo salar *(Atlantic salmon), *Oncorhynchus mykiss *(rainbow trout), *Osmerus mordax *(rainbow smelt), *Coregonus clupeaformis *(lake whitefish), *Salvelinus fontinalis *(brook trout), and *Thymallus thymallus *(grayling) [Table 2] were BLASTed against each other (evalue < 1e-35, hits > 100bp) and the results used to generate clusters of contigs. Bins of similar sequences, or clusters, were generated containing all contigs irrespective of species origin that had alignments with greater than 75% of the length of the shorter sequence and had greater than 70% identity in the overlapping regions (alignments consisted of ends-free alignment with scores of 2/-2/-5/-1 for match/mismatch/open gaps/extend gaps [43]. After the contigs had been grouped into clusters, the individual clusters were then further selected to only contain contigs that had mutually overlapping regions and all contig members were trimmed to the largest common alignment (same alignment parameters as above). A good alignment was considered to be greater than or equal to 300 bp in length with greater than 60% identity in the overlapping region. At this point, clusters that did not contain at least one sequence from each of the six target species were discarded. This resulted in a dataset of 78 clusters or gene sets. All gaps (and their corresponding positions in other sequences of the cluster) were removed, and the data within each gene set were bootstrapped 500 times. The PHYLIP package was used because it offers many different analysis methods, is freely available and is commonly used [44]. Distance matrices were computed for each bootstrapped dataset within each cluster using the F84 model of nucleotide substitution and Gamma-distributed rates of variation across sites with a coefficient of variation of 0.5 [44]. Neighbor-joining trees were then computed from each set of distance matrices and the set of resulting bootstrapped trees was used to derive a 70%-majority consensus tree [44]. The consensus trees were rooted with *Osmerus mordax*, and simplified by iteratively collapsing all pairs of leaf nodes having the same species and showing > = 98% similarity in the aligned portion of their sequences. Independently, maximum likelihood trees were generated for all 78 data sets using the default options with the Phylip program dnaml (transition/transversion ratio of 2.0, empirical base frequencies, constant rate variation among sites). A general evolutionary model was used for the 78 data sets because each set potentially consisted of a mixture of unidentified coding and non-coding data. All of the 78 ML trees were consistent with their 70%-consensus bootstrapped Neighbor-joining counterparts. EST accession numbers used to make contig consensus sequences, alignments and the 70% consensus trees are available [see Additional file 1] or online at the GRASP website [24].

### Microarray Clone selection

Starting from the existing GRASP 16 K cDNA microarray [24], additional clones were selected for representation on the following basis: a) the contig (Table 2) includes at least one clone that is on hand; b) the contig is of high quality with few conflicting positions, few singleton positions, no interior singleton positions (potential chimeric sites) and there are at least two clones in the contig (from at least 2 plates, and preferably from at least 2 libraries); c) if the contig is singleton then it must have a good BlastX hit (e-value < 1e-8) or other indication of orientation (eg. consistent poly(A) tail information); d) contig must have < = 94% identity to another sequence on the chip (the existing 16 K plus any new contig; not counting rainbow trout orthologs); and e) no tRNA, ribosomal, or mitochondrial sequences. We chose clone representatives within each contig based on: a) the reliability of the cDNA library and sequence; b) high similarity to consensus of contig (allow 20 bp at ends for poor trimming); c) reliable sequence from the 3'-end of contig and correct (3' -> 5') orientation; and d) ownership of clone.

### Microarray fabrication

The initial clones were robotically rearrayed from daughter glycerol stock 384-well plates into 96-well plates prefilled with 8% glycerol in 2XYT + ampicillin with a MicroGrid II-610 (Biorobotics, Cambridge, UK), incubated overnight at 37°C, and checked for uniform optical density. Plasmid inserts were PCR-amplified in a MJ Tetrad PTC-205 thermocycler (Bio-Rad, Hercules, CA, USA) by using 1.0 μL overnight culture, 0.3 μM M13/pUC forward primer (5'-CCCAGTCACGACGTTGTAAAACG-3'), 0.3 μM M13/pUC reverse primer (5'-AGCGGATAACAATTTCACACAGG-3'), 2 mM MgCl2, 10 mM Tris-HCl, 50 mM KCl, 200 μM dNTPs, 1U AmpliTaq (Roche Diagnostics, NJ, USA), and nuclease-free H2O (Qiagen, Valencia, CA, USA) to 100 μL. PCR conditions were as follows: 2 min at 95°C denaturation; 35 cycles of 30 sec at 95°C, 45 sec at 59°C, and 4 min at 72°C; and 7 min at 72°C. Hotstar taq (Qiagen) was used to amplify additional inserts (clone set 2) with an initial denaturation of 15 mins. Amplicon specificity and yield was analyzed by capillary electrophoresis using the HT DNA SE 30 LabChip on Caliper AMS 90 system (Zymark-Caliper Life Sciences, MA, USA). PCR products were robotically cleaned (Qiagen) and consolidated into 384-well plates, lyophilized by speed-vac, and resuspended in 20 μL 3× SSC plus 1.0 M betaine. All cDNAs (average printing concentration of 165 ng/ul [original inserts] and 100 ng/ul [new inserts]) were printed as single spots on Erie Aminosilane slides (Erie, Portsmouth, N.H., USA) with a Genetix QArraymax microarray printer (Genetix, New Milton, Hampshire, UK) or MicroGridII-610 printer (Biorobotics, Cambridge, UK). All clones and controls were distributed randomly on the array. Genetix aQu 65 um quill pins or Biorobotics 10 k quill pins in a 48-pin tool were used to deposit < 1.0 nL (0.1 ng cDNA) per spot onto the slide. The resulting microarrays have a 4 × 12 subgrid layout with 699 spots per subgrid, each spot having diameter and pitch of 90–130 and 160–190 μm, respectively. A 280-bp GFP (green fluorescent protein) cDNA was amplified from a GFP clone (BD Biosciences, Mountain View, CA, USA) by using the primers (5'-GAAACATTCTTGGACACAAATTGG-3') and (5'- GCAGCTGTTACAAACTCAAGAAGG-3'), and printed in subgrid corners to assist in placing on the grid. The slides were crosslinked in a UV Stratalinker 2400 (Stratagene, La Jolla, CA, USA) at 300 mJ. One slide every 20 to 30 slides was hybridized with labeled random 9-mer oligonucleotide (SpotQC, Integrated DNA Technologies, Coraville, IA, USA) and scanned using GenePix 4200AL scanner (Molecular Devices, Sunnyvale, CA, USA). Presence/absence, shape, signal intensity vs. background, diameter and DNA binding site capability were measured for each spot on the slide using files generated by Imagene software (BioDiscovery Inc., El Secundo, CA, USA). Position and description of flagged spots (spots absent or thought to be unusable during post hybridization analysis), sub-grid defects and other noticed irregularities are recorded. Two PCR fragments from each plate were randomly selected and sequenced to ensure correct matches to the original clone sequence in the EST database. For controls, Stratagene SpotReport Alien cDNA Array Validation system PCR products (Cat # 252550) composed of 10 unique PCR products are spotted five times on the array. Corresponding mRNA for these PCR products can be purchased from Stratagene. The alien mRNA spikes can be used to determine mRNA quality, cDNA synthesis efficiency, positive and negative hybridization control, normalization for dye differences and determination of hybridization consistency.

### Microarray hybridizations

The microarray experiments were designed to comply with MIAME guidelines. To minimize technical variability, all targets were synthesized in one round and hybridization experiments were conducted on slides from a single batch. Each hybridization experiment included dye-flips to compensate for cyanine fluor effects. Total RNA samples were quantified and quality-checked by spectrophotometer and agarose gel, respectively. All hybridization experiments were performed using the SuperScript III Indirect cDNA Labeling System kit and following manufacturers instructions (Invitrogen). Briefly, total RNA was reverse transcribed using an anchored oligo d(T)_20 _primer in cDNA synthesis reactions that incorporated aminoallyl- and aminohexyl-modified nucleotides. The modified cDNAs were then labeled with fluorescent Cy5 or Cy3 dye in reactions with the amino-functional groups in coupling buffer.

All microarrays were prepared for hybridization by washing 2 × 5 min in 0.1% SDS, washing 5 × 1 min in MilliQ H_2_O, and drying by centrifugation (520 g for 5 min in 50 ml conical tube). All slides were prehybridized in 5 × SSC, 0.1% SDS, 3% BSA for 1.5 h at 49°C. Arrays were briefly washed 3 × 20 sec in MilliQ H_2_O, then dried by centrifugation. A total of 200 ng of labeled cDNA with each fluor was applied to prewarmed microarrays in a formamide-based buffer (25% formamide, 4× SSC, 0.5% SDS, 2× Denhardt's solution) 16 h at 49°C. The arrays were washed 1 × 10 min at 49°C (2× SSC, 0.1% SDS), and then 2 × 5 min in 2× SSC, 0.1% SDS, 2 × 5 min in 1× SSC and 4 × 5 min in 0.1× SSC at room temperature, then dried by centrifugation.

### Microarray analyses

Fluorescent images of hybridized arrays were acquired immediately at 10 um resolution using ScanArray Express scanner (PerkinElmer). The Cy3 and Cy5 cyanine fluors were excited at 543 nm and 633 nm, respectively, at the same laser power (90%), with adjusted photomultiplier tube settings between slides to balance the Cy5 and Cy3 channels. Fluorescent intensity data was extracted from TIFF images using Imagene 5.6.1 software (Biodiscovery). Quality statistics were compiled in Excel from raw Imagene fluorescence intensity report files. The hybridization performance of labeled targets to salmonid features was assessed as a percentage of features bound from the numbers of AS and RT features passing a hybridization signal threshold. Signal threshold was defined by 2 standard deviations above the signal mean for the 3× SSC/betaine buffer spots. Outliers of buffer spots were removed based on the Median Absolute Deviation method [45] whereby elements with a test statistic value greater than 5 were removed. No transformations or normalizations were performed on these data. Only features deemed present by Imagene 5.6.1 (excluding marginal and absent values) were used for analyses.

## Authors' contributions

WSD, BFK conceived the project, analyzed data and wrote the paper. JL, NW, and RL performed much of the data analysis and KRVS, GAC, MB, AR, and CRM performed library preparation, microarray work, sequencing, data entry and analysis. RAH, and RM performed much of the DNA sequencing, and SB and JR coordinated PCR and printing microarrays. All authors have read and approved the general content of the manuscript.

## Supplementary Material

Additional file 1 **78 phylogenetic data sets and trees**. The data provided represent the EST sequences, alignments, and trees used in the phylogenetic analyses.
This file requires the "Tar" UNIX command to open it. Click here for file
